# Antibiotics for acute respiratory tract infections: a mixed-methods study of patient experiences of non-medical prescriber management

**DOI:** 10.1136/bmjopen-2016-013515

**Published:** 2017-03-15

**Authors:** Molly Courtenay, Samantha Rowbotham, Rosemary Lim, Rhian Deslandes, Karen Hodson, Katie MacLure, Sarah Peters, Derek Stewart

**Affiliations:** 1 School of Healthcare Sciences, Cardiff University, Cardiff, UK; 2 Menzies Centre for Health Policy, University of Sydney, Sydney, New South Wales, Australia; 3 The Australian Prevention Partnership Centre, Sax Institute, Sydney, New South Wales, Australia; 4 Reading School of Pharmacy, Reading, UK; 5 Cardiff School of Pharmacy and Pharmaceutical Sciences, Cardiff University, Cardiff, UK; 6 School of Pharmacy and Life Sciences, Robert Gordon University, Aberdeen, UK; 7 Manchester Centre for Health Psychology, School of Health Sciences, University of Manchester, Manchester, UK

**Keywords:** RESPIRATORY MEDICINE (see Thoracic Medicine)

## Abstract

**Objective:**

To (1) explore patients' expectations and experiences of nurse and pharmacist non-medical prescriber-led management of respiratory tract infections (RTIs), (2) examine whether patient expectations for antibiotics affect the likelihood of receiving them and (3) understand factors influencing patient satisfaction with RTI consultations.

**Design:**

Mixed methods.

**Setting:**

Primary care.

**Participants:**

Questionnaires from 120 patients and follow-up interviews with 22 patients and 16 nurse and pharmacist non-medical prescribers (NMPs).

**Results:**

Patients had multiple expectations of their consultation with 43% expecting to be prescribed an antibiotic. There was alignment between self-reported patient expectations and those perceived by NMPs. Patient expectations for non-antibiotic strategies, such as education to promote self-management, were associated with receipt of those strategies, whereas patient expectations for an antibiotic were not associated with receipt of these medications. ‘Patient-centred’ management strategies (including reassurance and providing information) were received by 86.7% of patients. Regardless of patients' expectations or the management strategy employed, high levels of satisfaction were reported for all aspects of the consultation. Taking concerns seriously, conducting a physical examination, communicating the treatment plan, explaining treatment decisions and lack of time restrictions were each reported to contribute to patient satisfaction.

**Conclusions:**

NMPs demonstrate an understanding of patient expectations of RTI consultations and use a range of non-antibiotic management strategies, particularly those resembling a patient-centred approach. Overall, patients' expectations were met and prescribers were not unduly influenced by patient expectations for an antibiotic. Patients were satisfied with the consultation, indicating that strategies used by NMPs were acceptable. However, the lower levels of satisfaction among patients who expected but did not receive an antibiotic indicates that although NMPs appear to have strategies for managing RTI consultations, there is still scope for improvement and these prescribers are therefore an important group to involve in antimicrobial stewardship.

**Strengths and limitations of this study**

- A mixed-methods approach with the inclusion of patient and non-medical prescriber (NMP) interviews allowed for triangulation of methods and validation of data as well as enabling a richer picture and greater insights into quantitative findings.
- NMPs were asked to approach consecutive patients to reduce bias, but they may have filtered out patients perceived to have experienced a less successful consultation; moreover, more dissatisfied patients may not have returned a questionnaire; therefore, the high levels of satisfaction may be an overestimate.
- Reducing antibiotic prescribing in patients with pre-existing chronic conditions is not always appropriate and as we did not specifically exclude those with chronic conditions, we therefore do not attempt to make any inferences about the appropriateness of antibiotic prescriptions.
- Data were self-reported, so they may not accurately reflect the consultation, as patients' recall of their expectations are likely to be influenced by what had occurred in the consultations.
- We do not know if non-responders of the patient questionnaire or patient interviews differed from responders, and so there may be bias within our data, such that those patients who were more satisfied may have agreed to participate.

## Introduction

Resistance to antibiotics is a major global health problem,^1^ ^2^ with overuse of antibiotics a key factor.^3^ ^4^ Conserving antibiotic sensitivity through the management of self-limiting respiratory tract infections (RTIs) without recourse to antibiotics is a priority.^1^ ^5–9^ Despite clear clinical guidelines^10^ and evidence that antibiotics are ineffective for coughs, colds and sore throats,^6^ have iatrogenic consequences and increase susceptibility to infection,^4^ many patients continue to ask for and receive antibiotics.^11^

Non-medical prescribers (NMPs), of whom there are 35 000 across the UK (mainly nurses but also some pharmacists and small numbers of allied health professionals including physiotherapists, podiatrists and radiographers), predominantly prescribe independently in general practice in primary care.^12–15^ High numbers prescribe for respiratory conditions^15^ and infections^12^ and they are therefore an important target group for antimicrobial stewardship efforts. While research has explored antibiotic prescribing for RTIs by general practitioners (GPs), there is scant evidence on NMPs' antibiotic prescribing for these conditions.^16^ Several factors have been identified that influence GPs' prescribing decisions,^17^ including perceived patient expectations,^18^ patient pressure,^19^ ^20^ diagnostic uncertainty and fear of complications,^21^ factors imposed by healthcare systems,^22^ and clinician characteristics.^22^ Evidence suggests that NMPs encounter similar concerns over diagnostic uncertainty, and are influenced by clinical guidelines, but are not unduly influenced by patient expectations for an antibiotic.^23^ NMPs view education and self-management as central components of their role and reportedly use these strategies to reduce reconsulting rates for similar future illnesses.^23^

Research has not yet explored patient experiences of NMP management of RTIs and how this impacts patient satisfaction. Therefore, the aims of the study were (1) to explore patients' expectations and experiences of nurse and pharmacist NMP-led management of RTIs, (2) to examine whether patient expectations for antibiotics affected their likelihood of receiving them and (3) to understand factors influencing patient satisfaction with RTI consultations.

## Methods

All participants gave informed consent (see online supplementary file) before taking part.

10.1136/bmjopen-2016-013515.supp1 supplementary file

### Design

A mixed-methods design was used in which qualitative and quantitative data were collected from patients and NMPs following consultations for RTIs.

### Recruitment

The sample was drawn from patients across Scotland, Wales and England presenting to an NMP with an RTI. Discussion with prescribing leads (with whom authors had collaborated previously) in one Health Board (HB) in Scotland and one Clinical Commissioning Group (CCG) in England identified both the HB and CCG to have NMPs working as substitutes for doctors (ie, providing services which otherwise would be provided by doctors alone),^24^ and responsible for providing first contact care and managing RTIs in primary care. An invitation to participate was sent via email by each lead to all NMPs in the HB (n=10) and CCG (n=15). Welsh HBs have not appointed prescribing leads and so graduates of the Cardiff University prescribing programme (n=7), working in primary care, were invited to participate. Interested NMPs across the three countries were provided with further information and those consenting (n=8 in England, n=5 in Scotland, n=4 in Wales) were invited to approach 10 consecutive patients (or parents/carer of child patients) presenting with a chief symptom consistent with an RTI.^10^ Patients deemed by NMPs to have insufficient English to provide informed consent, complete the questionnaire and participate in an interview were excluded. Consenting participants were invited to complete the questionnaire following their consultation and deposit it in a collection box at the practice reception prior to leaving. It was made clear that the NMP would not see or have access to the information within the questionnaire. Within the questionnaire was the option for patients to supply contact details if they wished to take part in a follow-up telephone interview. All those who did so were sent a participant information sheet and a consent form. Consenting patients took part in a telephone interview with a researcher (SR, TC or RL). All consenting NMPs also took part in a telephone interview with a researcher (RHML or TC). Data collection for the study took place between August 2014 and November 2015.

### Data collection

#### Patient questionnaire

A patient questionnaire developed for use by patients with RTIs, seen by GPs,^25^ was used to collect information on symptoms (earache, symptoms of nose/sinus, common cold, sore throat, cough), expectations and outcomes (ie, physical examination, information, reassurance; strategies within National Institute for Health and Care Excellence (NICE) guidelines for managing symptoms, ie, medication for pain relief, nose drops, cough medicine; antibiotics;^10^ referral to hospital physician) using yes/no response options. Satisfaction with various aspects of the consultation (amount of time on symptom, amount of information given, the content of information given, the proposed treatment, extent to which they felt they were taken seriously, overall satisfaction) was measured using a five-point Likert scale (1=very dissatisfied to 5=very satisfied). Demographic data (gender, age and country) were also gathered.

#### Interviews

Semistructured individual NMP and patient interviews used a topic guide informed by the literature,^23–26^ to explore patients’ reasons for their consultation, their expectations and experience. NMP interviews explored factors perceived to motivate patients to consult, and their experiences of managing RTIs. Demographic data collected from patients included gender, age, country, symptoms. Demographic data from NMPs included gender, time in post and country. Patient interviews took place 4–8 weeks following the consultation. All interviews were digitally audio-recorded and transcribed verbatim and any identifying information removed.

### Data analysis

Data sets were collected and analysed separately but in the same time frame, and qualitative analysis was informed by the quantitative findings.^27^ Quantitative findings from the patient questionnaire were used to examine associations between patient expectations for antibiotics, antibiotic prescriptions received and patient satisfaction with care received. Qualitative data provided more in-depth insight into patient and NMP experiences of RTI consultations and their perception of factors that influence satisfaction.

#### Questionnaires

Questionnaire data were analysed within SPSS V.17 (SPSS Inc. Released 2008. SPSS Statistics for Windows, Version 17.0. Chicago: SPSS Inc). Descriptive statistics were calculated for patient expectations, management strategies received and satisfaction with the consultation. Satisfaction data were categorised for analysis as ‘not satisfied’ (ie, very dissatisfied, dissatisfied, neither satisfied nor dissatisfied) and ‘satisfied’ (satisfied or very satisfied)*.* Fisher's exact test was used to explore associations between patient expectation, management strategies received and satisfaction with the consultation. An α of 0.05 was applied except where multiple analyses were conducted for the same outcome variable, in which case a Bonferroni correction (0.05/number of analyses) was applied to control for familywise error rate (ie, increased likelihood of type 1 error).

#### Semistructured interviews

Inductive thematic analysis was conducted on interview data, with emerging findings cross-checked against the results from the questionnaire.^28^ Initial coding and categorising of data were managed within NVivo V.10 using line-by-line coding. Master themes were identified (supported by data from each participant group), allowing identification of cross-cutting patterns and themes within and across the data. The thematic structure was revised through discussion with the wider research team. To increase the trustworthiness of the final analysis, each transcript was read and coded independently by two experienced qualitative researchers (SR and RHML). Differences in interpretation were resolved through discussion between coders and the wider research team. This is an established method to increase the trustworthiness of research at the interface of several disciplines.^26^ Saturation was achieved in that later interview data were able to be categorised within the existing coding frame without needing to add new codes.

The concurrent analysis of interview data from NMPs and patients, described above, allowed for examination of consistency of the findings emerging from the two data sets, with the questionnaire data providing a further point of reference for consistency and validity checking. This ongoing and iterative process of comparing the findings emerging from each of the data sets allowed for data triangulation, thereby confirming the accuracy of findings across patient and NMP interviews and offering a more holistic portrayal of the phenomenon under study.^28^ ^29^ Member validation (presentation of findings at a CCG NMP workshop) helped to establish trust of emerging analysis.^26^

## Results

Seventeen NMPs (16 nurses, 1 pharmacist) were recruited. All nurse prescribers were in general practices and the pharmacist prescriber was based in a community clinic managed by GPs. Recruited NMPs identified 160 eligible patients of whom 120 (75%; including 8 parents of a child patient) consented and completed the questionnaire. Twenty-two patients (including the parent of 1 child patient) and 16 NMPs took part in a telephone interview. Patient interviews lasted between 8 and 21 min (mean=14 min). NMP interviews lasted between 9 and 19 min (mean=13 min). Demographic characteristics for the patient (including symptoms) and NMP samples are presented in tables 1 and 2, respectively.

**Table 1:** Demographic data (n (%)) for patients in the questionnaire and interview samples

	Questionnaires (n=120)	Interviews (n=22)
Gender		
Male	33 (27.5%)	9 (40.9%)
Female	55 (45.8%)	13 (59.1%)
Age		
<25	32 (26.7%)	2 (9.1%)
26–35	18 (15.0%)	1 (4.5%)
36–45	12 (10.0%)	3 (18.2%)
46–55	16 (13.3%)	4 (13.6%)
56–65	37 (30.8%)	6 (31.8%)
>65	4 (3.3%)	2 (22.7%)
Symptoms		
Earache	16 (13.3%)	1 (4.5%)
Sinus	25 (20.8%)	1 (4.5%)
Sore throat	70 (58.3%)	4 (18.2%)
Cough	65 (54.2%)	9 (40.9%)
Cold	23 (19.2%)	6 (27.3%)
Other	30 (25.0%)	1 (4.5%)
Country		
England	75 (62.5%)	13 (59.1%)
Scotland	15 (12.5%)	2 (9.1%)
Wales	30 (25.0%)	7 (31.8%)

Owing to missing data from participants who chose not to disclose demographic information, the percentages do not always equal 100%.

**Table 2:** Demographic data (n (%)) for non-medical prescribers (n=16)

Gender	
Male	1 (5.9%)
Female	15 (94.1%)
Time in post (years)	
<2	4 (25.0%)
2–4	4 (25.0%)
5–11	8 (50.0%)
Country	
England	7 (47.1%)
Scotland	5 (29.4%)
Wales	4 (23.5%)

Owing to missing data from participants who chose not to disclose demographic information, the percentages do not always equal 100%.

### Patient expectations of RTI consultations

The questionnaire data revealed that while 43% of patients expected to receive an antibiotic (see table 4 in section ‘Associations between expected and received management strategies and satisfaction’), patients often had multiple expectations of the consultation, including information (58%), reassurance (52%), further physical examination (44%) and/or non-antibiotic medication: pain relief (19%), cough medicine (41%), nose drops (7%).

Qualitative data revealed that patients who expected an antibiotic were often influenced by their history of receiving antibiotics for similar symptoms or existing respiratory conditions, where having a back-up prescription was the norm in case symptoms worsened.

I’d already had a chest infection in May which I’d had antibiotics prescribed for and I knew that I was in a similar situation… (Patient 7)

Some patients reported the desire to ‘get better’ quickly, to receive self-management advice, or reassurance, as their motivation for consulting, rather than wanting an antibiotic. Patients also expected NMPs to conduct a physical examination.

Not to necessarily get a bit of paper with a prescription it's to get reassurance that somebody is understanding your problem your oxygen levels fine…your breathing your chest's fine, it's just, you know, a bit bad at night and you will get better. (Patient 13)

It was more to get better, so I could get back [to work] quickly. (Patient 21)

There was considerable alignment between patient expectations as perceived by NMPs and identified by patients themselves. NMPs recognised the influence of past experience and perceived the older generation as being more likely to expect an antibiotic. NMPs reported that for some patients, antibiotics were viewed as a ‘quick fix’.

Younger ones, they want the prescription because they want to go back to work, and they think, if you give them the prescription they will go back to work quicker. (NMP 10)

NMPs also recognised that patients often presented because of persistent symptoms that interfered with their lives or that they were concerned about, and thus expected reassurance and advice.

They just want to make sure, “…because my chest is always a problem, nurse, I just want to make sure I'm catching it early and I can't hear my own chest and I just want you to give it a listen to.” They just want reassurance often. (NMP 4)

### Management strategies employed within consultations

The questionnaire data revealed that 33% of patients received an antibiotic prescription (see table 3). Other management strategies recalled by patients were non-antibiotic medication (40%; ie, pain relief, nose drops, cough medicine), and strategies that we refer to as ‘patient-centred management’ (86.7%; ie, providing information, reassurance and/or further physical examination such as listening to the patient's chest and taking oxygen levels). Only a small number of patients reported that they were prescribed antibiotics without accompanying examination or advice. Moreover, twice as many patients who did not receive an antibiotic reported that they received patient-centred management strategies (58%) compared with those who received antibiotics (28%).

**Table 3:** Number of patients (n (%)) receiving various treatment combinations of antibiotics, patient-centred management and/or non-antibiotic medication

Treatment	n (%)
Antibiotics only	6 (5.0%)
Antibiotics+patient-centred management	23 (19.2%)
Antibiotics+non-antibiotic medication	0 (0.0%)
Antibiotics+patient-centred management+non-antibiotic medication	11 (9.2%)
Patient-centred management only	43 (35.8%)
Patient-centred management+non-antibiotic medication	27 (22.5%)
Non-antibiotic medication only	10 (8.3%)

*‘*Patient-centred management’ in this context refers to information, reassurance and further examination. ‘Non-antibiotic medication’ refers to pain relief, cough medication and nose drops.

Within the interviews, NMPs reported explaining ‘no antibiotic’ decisions, providing information about treatment, and directing patients to information leaflets and websites. Time to educate patients was described as crucial for managing expectations. Clear communication was recognised as requiring time and empowering patients to take control of their illness and preventing unnecessary future consultations or expectation for antibiotics.

If you take time to discuss things with them, give them a lot of verbal information, and then back it up with something printed then people really appreciate that. (NMP 15)

Although most NMPs conducted physical examinations to aid diagnosis, some used them as a means of reassuring patients and/or as ‘evidence’ to justify their non-antibiotic management approach. This was perceived as resulting in patients being reassured and more accepting of NMPs' decision not to prescribe antibiotics.

I think it reassured the patient, even though I didn't feel myself it was necessary. Because sometimes when you look at a patient you can see yourself that, you know they're fairly a-symptomatic. But I would always do, you know, basic examinations to reassure them. (NMP 12)

NMPs also offered patients the opportunity to reconsult, and believed that this allayed patients' concerns. One NMP made follow-up calls to some patients highlighting that eased patients' concerns about their condition and the lack of antibiotics.

I will say, well look, I will give you a ring on Friday to see how you are doing and then we know you'll be covered for the weekend and that helps a lot, particularly with the older ones. (NMP 10)

Finally, some NMPs used delayed prescribing as a strategy for patients with asthma or chronic obstructive pulmonary disease due to the risks associated with these patients.

I think they quite like that option, it's all about patient information, and if as a clinician, you don't feel the need for antibiotics, but you know maybe it's a long weekend or something,…so that they have a plan. So you know if things deteriorate and spitting turns green, they have the antibiotics. (NMP 5)

Patient interviews supported NMP claims about strategies used within the consultation. Patients reported receiving physical examinations, including having their blood oxygen levels checked, chest listened to and lung function assessed using a peak flow meter, receiving advice on self-help measures, reassurance about their symptoms and further information about their condition, and being offered a repeat consultation if their symptoms persisted. Finally, concerning antibiotics, patients valued NMPs providing an explanation and rationale for prescribing decisions.

She examined my chest…took my temperature and did everything that she should have done you know my blood pressure and everything… (Patient 12)

She gave me good advice and said by the time she has given me anything to treat it with that is possibly an antibiotic or something the problem would have got better… (Patient 13)

### Associations between expected and received management strategies and satisfaction

#### Association between expected and received management

As shown in table 4, those who expected non-antibiotic strategies (non-antibiotic medications, information, reassurance and further examination) were significantly more likely to receive them than those who did not, and vice versa. However, expectations for antibiotics were not associated with patients receiving an antibiotic, and a substantial proportion of patients did not receive antibiotics, even when these were expected (see table 4).

**Table 4:** Associations between expected and received respiratory tract infection management strategies (n (%)) calculated using Fisher's exact test

Expectations	Received	Did not receive	p Value
	n (%)	n (%)	
Antibiotics			
Expected	23 (46.0%)	27 (54.0%)	
Did not expect	16 (24.6%)	49 (75.4%)	0.019
Pain relief			
Expected	10 (43.5%)	13 (56.5%)	
Did not expect	4 (4.1%)	93 (95.9%)	<0.001*
Nose drops			
Expected	3 (37.5%)	5 (62.5%)	
Did not expect	4 (3.6%)	107 (96.4%)	0.006*
Cough medication			
Expected	27 (55.1%)	22 (44.9%)	
Did not expect	7 (10.0%)	63 (90.0%)	<0.001*
Information			
Expected	68 (97.1%)	2 (2.9%)	
Did not expect	24 (48.0%)	26 (52.0%)	<0.001*
Reassurance			
Expected	58 (93.5%)	4 (6.5%)	
Did not expect	27 (46.6%)	31 (53.4%)	<0.001*
Further examination			
Expected	44 (84.6%)	8 (15.4%)	
Did not expect	26 (38.8%)	41 (61.2%)	<0.001**

Following a Bonferroni correction, the adjusted significance level is p<0.007.

Owing to missing data from participants who chose not to complete all sections of the questionnaire, the number of participants does not always equal 120.

*Used to denote significant results.

#### Patient satisfaction

The questionnaire data revealed that 96% of patients were satisfied or very satisfied with the consultation overall, and most were satisfied or very satisfied with specific aspects of the consultation (see table 5).

**Table 5:** Patient satisfaction (n (%)) with each aspect of the consultation

	Very dissatisfied	Dissatisfied	Neither satisfied nor dissatisfied	Satisfied	Very satisfied
	n (%)	n (%)	n (%)	n (%)	n (%)
Amount of time	2 (1.7%)	0 (0.0%)	7 (5.8%)	16 (13.3%)	95 (79.2%)
Amount of information	2 (1.7%)	0 (0.0%)	3 (2.5%)	17 (14.2%)	98 (81.7%)
Content of information	2 (1.7%)	1 (0.8%)	3 (2.5%)	15 (12.5%)	99 (82.5%)
Proposed treatment	2 (1.7%)	3 (2.5%)	11 (9.2%)	9 (7.5%)	95 (79.2%)
Extent taken seriously	2 (1.7%)	0 (0.0%)	1 (0.8%)	13 (10.8%)	104 (86.7%)
Overall	2 (1.7%)	0 (0.0%)	3 (2.5%)	13 (10.8%)	102 (85.0%)

The final stage of the quantitative analysis considered whether the alignment between patient expectation and receipt of the various management strategies was associated with patient satisfaction (see table 6). The alignment between expectation and receipt of antibiotics was significantly associated with patient satisfaction with treatment (p<0.001), such that only 59% of those who expected but did not receive antibiotics were satisfied with the treatment received. However, the alignment between expectation and receipt of antibiotics was not associated with overall satisfaction with the consultation. The alignment between expected and received strategy was not associated with overall satisfaction for any other management strategy (non-antibiotic medications or patient-centred management; all p>0.01), and only information was associated with satisfaction with treatment (p=0.006), most likely due to patients reporting high levels of satisfaction regardless of expected and received strategy.

**Table 6:** Associations between patient satisfaction and alignment of patient expectation and receipt of management strategies

	Satisfied with treatment	Satisfied overall
	n (%)	n (%)
Antibiotics		
Expected and received	23 (100%)	23 (100%)
Expected and not received	16 (59%)	24 (89%)
Not expected and received	16 (100%)	16 (100%)
Not expected and not received	46 (94%)	49 (100%)
p Value	<0.001*	0.032
Non-antibiotic medications		
Expected and received	35 (88%)	37 (93%)
Expected and not received	18 (69%)	24 (92%)
Not expected and received	7 (88%)	8 (100%)
Not expected and not received	44 (96%)	46 (100%)
p Value	0.019	0.183
Information		
Expected and received	59 (87%)	64 (94%)
Expected and not received	2 (100%)	2 (100%)
Not expected and received	17 (71%)	23 (96%)
Not expected and not received	26 (100%)	26 (100%)
p Value	0.006*	0.700
Reassurance		
Expected and received	49 (84%)	54 (93%)
Expected and not received	4 (100%)	4 (100%)
Not expected and received	23 (85%)	26 (96%)
Not expected and not received	28 (84%)	31 (100%)
p Value	*0.*776	0.426
Examination		
Expected and received	40 (91%)	43 (98%)
Expected and not received	6 (75%)	8 (100%)
Not expected and received	22 (85%)	24 (92%)
Not expected and not received	36 (85%)	40 (95%)
p Value	0.499	0.677

Following a Bonferroni correction, the adjusted significance level is p<0.01.

Owing to missing data from participants who chose not to complete all sections of the questionnaire, the number of participants does not always equal 120.

*Used to denote significant results.

The qualitative data support these findings, with NMPs perceiving patients to be satisfied with their consultations. Conducting a thorough physical examination, clearly communicating the treatment plan and explaining treatment decisions were reported to contribute to patient satisfaction, as did the absence of time restrictions on consultations.

If you give a very good physical assessment, and then go through your findings with them, they are quite happy to not have a prescription, most of the time. (NMP 5)

The patient interviews confirmed that NMPs were perceived to be thorough and patients appreciated the additional time NMPs spent with them (compared with GPs). Patients felt this made them feel they were being taken seriously which contributed to their satisfaction.

It was great, she explained everything really clearly and listened to my chest,…The whole process took some twenty minutes, it was very thorough. (Patient 21)

She's very friendly, she's very open she doesn’t make you feel that you’re making a fuss unnecessarily, she chats to you as if you are an individual. You don’t feel there's a time limit… (Patient 7)

## Discussion

### Statement of principal findings

This is the first study to look at links between NMP management and patient experience of RTI consultations. Patients (primarily female, aged between 56 and 65 years) had multiple expectations of their consultation with less than half expecting an antibiotic. There was alignment between patient expectations as perceived by NMPs and identified by patients themselves and most patients received patient-centred management. A prescription for an antibiotic was not determined by a patient's expectation for an antibiotic. Although there were high levels of satisfaction following NMP consultations, a substantial proportion of patients who expected but did not receive antibiotics reported dissatisfaction with their treatment. Conducting a physical examination, communicating the treatment plan, explaining treatment decisions and lack of time restrictions each contributed to satisfaction and led patients to feel they were being taken seriously and treated by a healthcare expert.

### Strengths and weaknesses

A mixed-methods approach and the inclusion of patient and NMP interviews allowed for triangulation of methods and validation of data as well as enabling a richer picture and greater insights into quantitative findings. However, although asked to approach consecutive patients, NMPs may have filtered out patients perceived to have experienced a less successful consultation. While the questionnaire response rate was 75%, we do not know if non-responders differed from responders as we have no comparison data. Similarly, since we only had the contact details of those wishing to participate in a follow-up interview, comparisons between those who did and did not participate was not possible. There may, therefore, be bias within our data, for example, more satisfied patients may have agreed to participate. Reducing antibiotic prescribing in patients with pre-existing chronic conditions is not always appropriate and as we did not exclude patients with such conditions, we therefore do not attempt to make any inferences about the appropriateness of antibiotic prescriptions. Further, since our study included both adult and child patients, we acknowledge that parental expectations regarding care of their child may be distinct from adult expectations about their own care. While our sample of parents was not large enough to explore these differences (only 6% of questionnaire sample and 5% of interview sample were parents of a child patient), this is an area that warrants future research to better understand the strategies needed when dealing with different types of consultations. Finally, patient expectations are influenced by social gradient;^30^ however, we do not have the data to consider this within our sample and therefore do not know the extent to which these patients actively sought out NMPs.

Data were self-reported, and therefore may not accurately reflect the consultation, as patients' recall of their expectations may be influenced by what had occurred in the consultations. Furthermore, patient interviews took place 4–8 weeks following the consultation which may also have affected recall. However, the close alignment between the questionnaire responses and both the patient and NMP interviews indicates that this was not overly problematic. Length of NMP and patient interviews was short; however, data saturation was achieved.

### Comparison with other studies

The findings confirm that patients have multiple expectations of RTI consultations with NMPs, including information and support for self-management, antibiotics and non-antibiotic medication for symptom relief. This is in line with the findings from research on patient expectations of RTI consultations with GPs.^30^ The alignment between the expectations reported by patients and perceived by NMPs suggests that NMPs actively explored patient expectations, supporting previous findings that nurse prescribers are skilled at eliciting patient expectations.^31^

Expectations for antibiotics were not associated with patients receiving them, suggesting NMPs are not necessarily influenced by patient expectations for antibiotics. Although sample numbers in the current study are much smaller than in those studies that have explored the influence of patient expectations on the prescribing behaviour of GPs, such studies report that GPs are significantly more likely to prescribe antibiotics if they perceive that the patient expects one.^19^ ^30^ It is important to note, however, that expectation of an antibiotic is not necessarily unwarranted, such that in some instances patients expect antibiotics when it is indeed appropriate for them to receive one (eg, patients with chronic respiratory conditions).

Patient-centred management strategies were received by most patients irrespective of whether they received an antibiotic. Patients reported being taken seriously and having their concerns listened to, reassurance and advice provided, treatment plans discussed, and treatment decisions explained and shared. This approach encouraged patients to share personal information, raise their concerns and seek clarification about their condition or treatment. This aligns with studies that have explored patient experiences of NMP management of long-term conditions in which the adoption of patient-centred principles have been reported to improve patient understanding of treatment and conditions and improve self-care.^31^ ^32^

Regardless of the management strategy employed, high levels of satisfaction were reported for all aspects of the consultation. These results indicate that by adopting a patient-centred approach, NMPs addressed patient expectations and concerns, maintaining a high level of satisfaction. This aligns with studies that have investigated satisfaction following a patient-orientated intervention designed to reduce antibiotic prescribing, which found that regardless of whether patients received an antibiotic, most patients were satisfied.^33^ However, it should be noted that there were lower levels of satisfaction among patients who expected but did not receive an antibiotic (only 59% of these patients were satisfied with the treatment). Therefore, although NMPs appear to have strategies for managing RTI consultations, there is still scope for improvement.

### Meaning of the study: possible explanations and implications for clinicians and policymakers

Interventions designed to address the complex behaviour of, and between, prescriber and patients should also be informed by an understanding of the experiences of the large numbers of NMPs working in primary care and these groups should be involved in efforts to improve antimicrobial stewardship. It follows that there are opportunities for interprofessional learning with regard to the management of consultations for RTIs. Patient-centred care is a major feature of the nurse and pharmacist role,^34^ ^35^ and a focus in nursing and pharmacy curricular.^36^ ^37^ Interventions that correspond to a framework of patient-centred care and promotion of shared decision-making have been reported to significantly reduce antibiotic prescribing for RTIs.^38^

### Unanswered questions and future research

Systematic reviews report that patient satisfaction is higher when nurses, as opposed to doctors, provide first contact care, with longer consultations of nurses cited as a possible reason for this.^24^ ^39^ ^40^ An important next step would therefore be in-depth comparison of NMP and GP RTI consultations to understand similarities and differences in experiences, challenges and management strategies. As part of this, it would be valuable to explore differences in the patients who request or are referred to GPs and NMPs, for example, whether local triage processes result in NMPs seeing ‘less complex’ cases. This would provide insights into the specific kinds of support that NMPs and GPs require in terms of ensuring appropriate antibiotic prescribing.

The results of this and other studies indicate that NMPs and GPs have similar experiences of these consultations and that there is scope for interprofessional learning to improve the management of these conditions while maintaining satisfaction. Our results suggest that patient-centred strategies were valued by patients and may be a focus for interventions. Previous work has shown that these interventions are useful for GPs, so a next step would be to consider their utility with NMPs.

## Conclusion

NMPs demonstrate an understanding of patient expectations of RTI consultations and use a range of non-antibiotic management strategies, particularly in terms of taking a patient-centred approach. Overall, patients' expectations were met and prescribers were not unduly influenced by patient expectations for an antibiotic. Patients were satisfied with the consultation, indicating that strategies used by NMPs were acceptable. However, the lower levels of satisfaction among patients who expected but did not receive an antibiotic indicate that although NMPs appear to have strategies for managing RTI consultations, there is still scope for improvement and these prescribers are therefore an important group to involve in antimicrobial stewardship.
