# Supplementary material for: Antibiotics for acute respiratory tract infections: a mixed-methods study of patient experiences of non-medical prescriber management
Source: BMJ Open. 2017 Mar 15;7(3):e013515. doi: 10.1136/bmjopen-2016-013515 (PMC5353344; doi:10.1136/bmjopen-2016-013515)
Supplement: supplementary file [file bmjopen-2016-013515supp.pdf]

School of Healthcare Sciences,
College of Biomedical and Life Sciences,
Cardiff University,
Cardiff CF24 OAB

Consent Form

The prescription of antibiotics for acute respiratory tract symptoms: Interviews about patients' expectations, management by non-medical prescribers, and patient satisfaction

- I the undersigned voluntarily agree to take part in the above study ☐
- I have read and understood the Information Sheet provided. I have been given a full explanation by the investigators of the nature, purpose, location and likely duration of the study, and of what I will be expected to do. I have been given the opportunity to ask questions on all aspects of the study and have understood the advice and information given as a result. ☐
- I agree to comply with any instruction given to me during the study and to co-operate fully with the investigators. ☐
- I understand that I am free to withdraw from the study at any time without needing to justify my decision and without prejudice. ☐
- I confirm that I have read and understood the above and freely consent to participating in this study. I have been given adequate time to consider my participation and agree to comply with the instructions and restrictions of the study. ☐

Name of volunteer (BLOCK CAPITALS) ....

Signed ....

Date ....

Name of researcher/person taking consent (BLOCK CAPITALS) ……..

Signed ....

Date ……………………………………..
